# Mir-338-3p targeting THBS1 attenuates glioma progression by inhibiting the PI3K/Akt pathway

**DOI:** 10.1186/s13062-023-00443-0

**Published:** 2024-01-24

**Authors:** Lianglei Jiang, Ting Fang, Tingting Hu, Jun Feng, Pengfei Yan

**Affiliations:** Department of Neurosurgery, , Union Hospital, Tongji Medical College, Huazhong University of Science and Technology , 430022 Wuhan, China

**Keywords:** Glioma, miR-338-3p, PI3K/Akt, THBS1

## Abstract

**Background:**

Glioma is a brain tumor with high morbidity and mortality rates. Understanding its molecular pathogenesis can provide targets and therapeutic strategies for glioma treatment. miR-338-3p represses tumor growth in several cancers, including glioma. Thus, this study aimed to identify the regulatory effects of miR-338-3p/phosphoinositide 3-kinase (PI3K)/Akt/thrombospondins 1 (THBS1) on glioma progression.

**Materials and methods:**

Quantitative reverse transcription polymerase chain reaction and western blotting were performed to evaluate the levels of miR-338-3p, THBS1, and PI3K/Akt phosphorylation-related proteins. TargetScan software predicted that miR-338-3p targeted THBS1. This was confirmed by performing the dual-luciferase assay. Wound-healing and cell-counting-kit-8 experiments were performed to analyze how THBS1 and miR-338-3p affect the ability of glioma cells to migrate and proliferate. The effect of miR-338-3p on tumorigenicity in mice was also analyzed.

**Results:**

miR-338-3p downregulation was observed in gliomas, whereas THBS1 showed the opposite trend. By suppressing the PI3K/Akt signaling pathway activation, miR-338-3p overregulated the ability of glioma cells to migrate and proliferate in vitro. Additionally, miR-338-3p inhibited the development of glioma tumors in vivo. Moreover, miR-338-3p directly targeted THBS1. THBS1 overexpression promoted glioma cell migration and proliferation by increasing PI3K/Akt phosphorylation. Nonetheless, miR-338-3p overregulation alleviated the effects of THBS1 overexpression.

**Conclusion:**

The miR-338-3p/PI3K/Akt/THBS1 regulatory axis can modulate the progression of glioma cell proliferation and migration; thus, it can be considered a therapeutic biomarker.

**Supplementary Information:**

The online version contains supplementary material available at 10.1186/s13062-023-00443-0.

## Highlights

MiR-338-3p is downregulated in glioma.

Overregulating miR-338-3p inhibits glioma progression.

THBS1 was upregulated in glioma and promotes glioma progression.

Overexpressed miR-338-3p targeting THBS1 attenuates glioma progression by PI3K/Akt pathway.

## Introduction

Glioma is a brain tumor derived from the neuroepithelium. It accounts for 30% of all primary brain tumors and exhibits significant morbidity and mortality rates for the tumor sites as well as frequent invasive growth [1]. In recent years, several studies have been conducted to elucidate the molecular pathogenesis of gliomas. Currently, surgery, radiotherapy, and chemotherapy are widely used in the clinic, but these methods cannot mitigate the malignant progression of gliomas [2, 3]. Hence, an improved understanding of its molecular pathogenesis is beneficial for the identification of molecular targets and delineation of therapeutic strategies.

MicroRNAs (miRNA), with 18–22 nucleotides in length, are one of the most abundant non-coding RNA families. They can be found in humans, animals, and plants and play a role in physiological and pathological processes by regulating RNA silencing and post-transcriptional processes [4]. An in-depth study of miRNAs has reported that they negatively modulate mRNA stability and translation [5]. Dysregulated miRNAs play essential roles in tumor diagnosis, regulating tumor growth and metastasis, and tumor prognosis [4]. miR-338-3p inhibits tumor development in several cancers, including glioma [6, 7]. Shang et al. [6] reported that miR-338-3p was downregulated in glioma cells, whereas miR-338-3p overregulation inhibited cell proliferation and promoted apoptosis. miR-338-3p is sponged by the long noncoding RNA LINC00689 and targets PKM pyruvate kinase M1/2 in glioma regulation, including growth, metastasis, and glycolysis [7]. However, studies on the role of miR-338-3p in gliomas are scarce.

Thrombospondins 1 (THBS1) is an extracellular matrix protein that affects diverse cellular activities and has been confirmed as a therapeutic biomarker for primary myelofibrosis [8]. THBS1 is highly expressed in breast cancer, oral squamous carcinoma, and lymphoma and enhances cancer progression by promoting tumor proliferation and metastasis [9–11]. A previous study showed that THBS1 knockdown inhibited glioma tumorigenesis by regulating the focal adhesion kinase/Akt signaling pathway [12]. However, whether THBS1 can be regulated by an miRNA that participates in glioma tumorigenesis has not yet been reported.

The phosphoinositide 3-kinase (PI3K)/Akt pathway is one of the most common pathways involved in tumorigenesis [13–15]. miRNAs that target their target genes to regulate the PI3K/Akt pathway have been reported in gliomas. For example, miR-24, which is highly expressed in gliomas, accelerated glioma cell growth by targeting caudal-type homeobox 1 and activating the PI3K/Akt pathway [16]. Furthermore, miR-3918 targeted epidermal growth factor receptor and inhibited the proliferative and migratory capacities of glioma cells by suppressing the activation of the PI3K/Akt pathway [17]. However, it remains unclear how miR-338-3p modulates glioma progression by targeting its target genes to regulate the PI3K/Akt pathway.

Therefore, we identified miR-338-3p gene targets and explored their roles in glioma progression via the PI3K/Akt pathway. Our present study makes a new attempt to reveal how the miR-338-3p/PI3K/Akt/THBS1 axis functions in gliomas. This molecular mechanism provides a complex regulatory axis that possesses the potential of a therapeutic biomarker for gliomas.

## Materials and methods

### Clinical samples collection

Glioma (N = 31) and paired adjacent normal (N = 31) tissues were collected from patients in our hospital. None of the patients received chemoradiotherapy before their surgeries. At least three pathologists validated the tissue samples as gliomas. Our hospital committee approved the present study. Written informed consent was obtained from all patients.

### Cell culture and transfections

Human glioma cells (HS683, A172, and U251) and primary normal human astrocytes (NHA) were provided by KeyGEN Biotech (Nanjing, China). All cells were cultured in Dulbecco’s modified Eagles Medium containing 10% fetal bovine serum (Gibco, Carlsbad, CA) and then maintained at 37°C in an environment with 5% CO_2_.

RiboBio (Shanghai, China) designed and synthesized agomiR-338-3p, miR-338-3p mimic, agomiR negative control (agomiR-NC), and mimic-NC. THBS1 sequences were subcloned into a plasmid vector (pcDNA3.1) with an empty vector as the NC. Lipofectamine 2000 (Invitrogen, Carlsbad, CA, USA) was used to introduce the mimics and vectors into U251 and A172 cells. After 48 h of transfection, the efficiency of the transfections was determined by performing quantitative polymerase chain reaction (qPCR).

### qPCR

Total RNA was extracted using the TRIzol reagent (Invitrogen). The expression of miR-338-3p and THBS1 was evaluated by performing qPCR. High-Capacity cDNA Reverse Transcription Kit (Applied Biosystems, USA) was used to reverse transcribe the total RNA to cDNA. The relative mRNA expression levels of THBS1 and miR-338-3p were assessed using HiScript II One Step qPCR SYBR Green Kit (#Q221) and miRNA Universal SYBR qPCR Master Mix (#MQ101), purchased from Vazyme Biotech Co., Ltd. (Nanjing, China). The relative expression of THBS1 and miR-338-3p was normalized against that of U6 and glyceraldehyde-3-phosphate dehydrogenase (GAPDH) and computed by adopting the 2^−△△Ct^ method. Genewiz (Nanjing, China) was used to synthesize miR-338-3p and THBS1 primers. The primers used are listed in Table 1.

**Table 1:** Real-time PCR primer sequences

Gene name	Sequence
miR-338-3p	Forward 5’-CGCGTCCAGCATCAGTGATT-3’ Reverse 5’-AGTGCAGGGTCCGAGGTATT-3’
THBS1	Forward 5’-AGACTCCGCATCGCAAAGG-3’ Reverse 5’-TCACCACGTTGTTGTCAAGGG-3’
GAPDH	Forward 5’-ATGGGGAAGGTGAAGGTCG-3’ Reverse 5’-TTACTCCTTGGAGGCCATGTG-3’

### Cell proliferation and migration

Cell proliferation analysis was performed using cells transfected with empty vectors, mimic-NC, THBS1-OE, miR-338-3p mimic, or THBS1-OE + miR-338-3p mimic. A cell counting kit-8 (CCK-8) assay was performed to assess cell viability. Every well on 96-well plates was inoculated with 2 × 10^4^ cells that had been transfected and pre-cultured for 24 h in a 5% CO_2_, 37°C environment. Then, CCK-8 solution (10 µL) was added to every well before the cells were maintained for two more hours at 37°C. A microplate reader (LabX, Switzerland) was used to analyze absorbance at 450 nm at the time points 0, 24 h, 48 h, and 72 h.

A wound-healing experiment was conducted to evaluate cell migration. We inoculated 3 × 10^4^ cells/well on 6-well plates and subsequently cultured them for 12 h. Thereafter, the cell layer was wounded with a sterilized 10 µL pipette tip before culturing the cells for 24 h in a medium without serum. Finally, the wounded area was photographed at the 0th and 24th hours, and the scratch areas were calculated.

### Western blotting

Proteins in the cells were isolated using the pre-cooled radioimmunoprecipitation assay lysis buffer (CWBIO, China), quantitated using a BCA protein assay kit (#P0011; Beyotime, Shanghai, China), and separated by performing 12% sodium dodecyl sulfate–polyacrylamide gel electrophoresis. Thereafter, the separated proteins were moved to polyvinylidene fluoride membranes, blocked using 5% non-fat milk for an hour at 25°C, and maintained at 4 °C overnight with primary antibodies sourced from Cell Signaling Technology, Inc. The primary antibodies were directed against the following target proteins: GAPDH (1:1,000, #5174), THBS1 (1:1000, #37,879), PI3K (1:1,000, #4257), phosphorylated (p)-PI3K (1:1,000, #17,366), Akt (1:1,000, #9272), and p-Akt (1:1,000, #4060). After incubation, the membranes were washed before another round of incubation for 2 h at 37°C with horseradish peroxidase-linked secondary antibodies (1:50,000, ab6721, Abcam). Finally, ECL Kit (Cat. #34,096) was used to visualize the membranes.

### Dual luciferase-reporter analysis

The miR-338-3p binding sequence on THBS1 was amplified and cloned into the psiCHECK2 vectors (Promega, Madison, WI, USA) to construct wild-type THBS1 (WT-THBS1), a THBS1 reporter. Additionally, a mutated THBS1 (Mut-THBS1) with a mutated target site (psiCHECK2-Mut) was constructed. Next, a combination of miR-338-3p/NC and WT/mut-THBS 1 was transfected into U251 and A172 cells. Twenty-four hours later, Dual-Luciferase Reporter Gene Assay Kit (Beyotime, Shanghai, China) was used to analyze luciferase signals.

### Xenograft Tumor analysis

Animal experiments were approved by our hospital’s ethics committee and were performed in accordance with the NIH Guide for the Care and Use of Laboratory Animals. For the tumor burden analysis in vivo, four-week-old BALB/c nude mice sourced from Charles River were used. The animals were injected with 2 × 10^7^ U251 cells harboring either agomiR-NC or agomiR-338-3p. They were housed, maintained at room temperature, and provided food and water *ad libitum*. Tumor volume was measured as described by Qian et al. [18], and data were collected every 4 days. The mice were sacrificed after 28 days by injecting them with 100 µL pentobarbital (i.p.). The tumors were photographed and weighed at the end of the experiment.

### Statistical analysis

Comparison analyses were carried out by performing one-way analysis of variance or Student’s *t*-test in GraphPad Prism 8.0 (CA, USA), depending on the number of groups. Comparison analyses were performed on the clinical samples using paired Student’s *t*-test. The data were presented as the mean ± standard deviation. Spearman’s correlation analysis showed the association between THBS1 and miR-338-3p expression. Statistical significance was set at *P* < 0.05.

## Results

### miR-338-3p was downregulated in glioma, and overregulated miR-338-3p inhibited tumor cell proliferation and migration by suppressing the PI3K/Akt phosphorylation

**Fig. 1:**
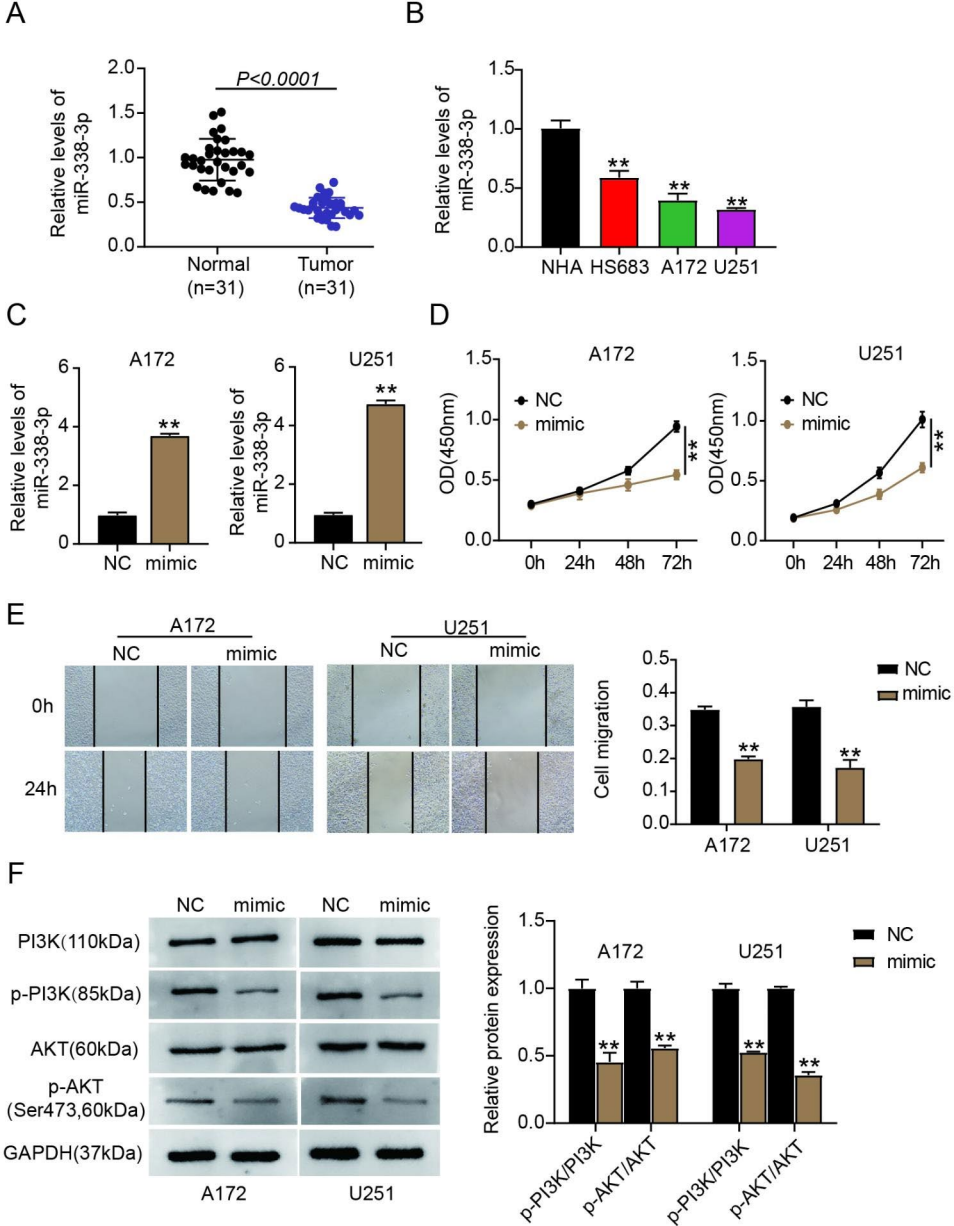
miR-338-3p was downregulated in glioma, and miR-338-3p overregulation repressed the migration and proliferation of tumor cells by suppressing PI3K/Akt phosphorylation. (**A**) miR-338-3p expression was assessed in normal and glioma samples by performing qPCR. (**B**) miR-338-3p levels were assessed in primary normal human astrocytes (NHA) and glioma cells (HS683, U251, and A172) via qPCR. ^**^P < 0.001 vs. NHA. (**C**) miR-338-3p expression was determined in U251 and A172 cells harboring either mimic-NC (NC) or miR-338-3p mimic (mimic). ^**^P < 0.001 vs. NC. (**D, E**) CCK-8 (**D**) and wound-healing (**E**) assays were performed in U251 and A172 cells with NC or mimic transfection to assess the abilities of the cells to proliferate and migrate. ^**^P < 0.001 vs. NC. (**F**) Levels of phosphorylated PI3K and AKT proteins in U251 and A172 cells with NC or miR-338-3p transfection were determined by performing western blotting. ^**^P < 0.001 vs. NC

To explore the mechanism by which miR-338-3p regulates gliomas, its regulatory trends were assessed. The qPCR analysis revealed considerably downregulated miR-338-3p expression in tumors relative to that in normal tissues (*P* < 0.0001; Fig. 1A). This was consistent with the results of the in vitro analysis, in which the glioma cells (HS683, U251, and A172) showed lower miR-338-3p expression than did NHA cells (Fig. 1B). Because U251 and A172 cells exhibited the lowest miR-338-3p expression, they were selected for in vitro functional experiments. The efficiency of the miR-338-3p mimic was validated by qPCR and is illustrated in Fig. 1C. Transfection with the miR-338-3p mimic markedly increased miR-338-3p levels. As indicated in Fig. 1D and 1E, compared with mimic-NC, miR-338-3p mimic transfection (*P* < 0.001) reduced the proliferative and migratory abilities of the cells. To study the role of the PI3K/Akt pathway in gliomas, the expression of p-Akt (Akt phosphorylation site Ser473) and p-PI3K were assessed. Compared with the miR-NC (NC) group, the expression of p-Akt and p-PI3K was notably downregulated in U251 and A172 cells transfected with the miR-338-3p mimic (*P* < 0.001; Fig. 1F). Overall, our findings indicated that miR-338-3p overexpression impeded the migration and proliferation of glioma cells by decreasing PI3K/Akt phosphorylation.

### Mir-338-3p overregulation inhibited glioma Tumor growth in vivo

**Fig. 2:**
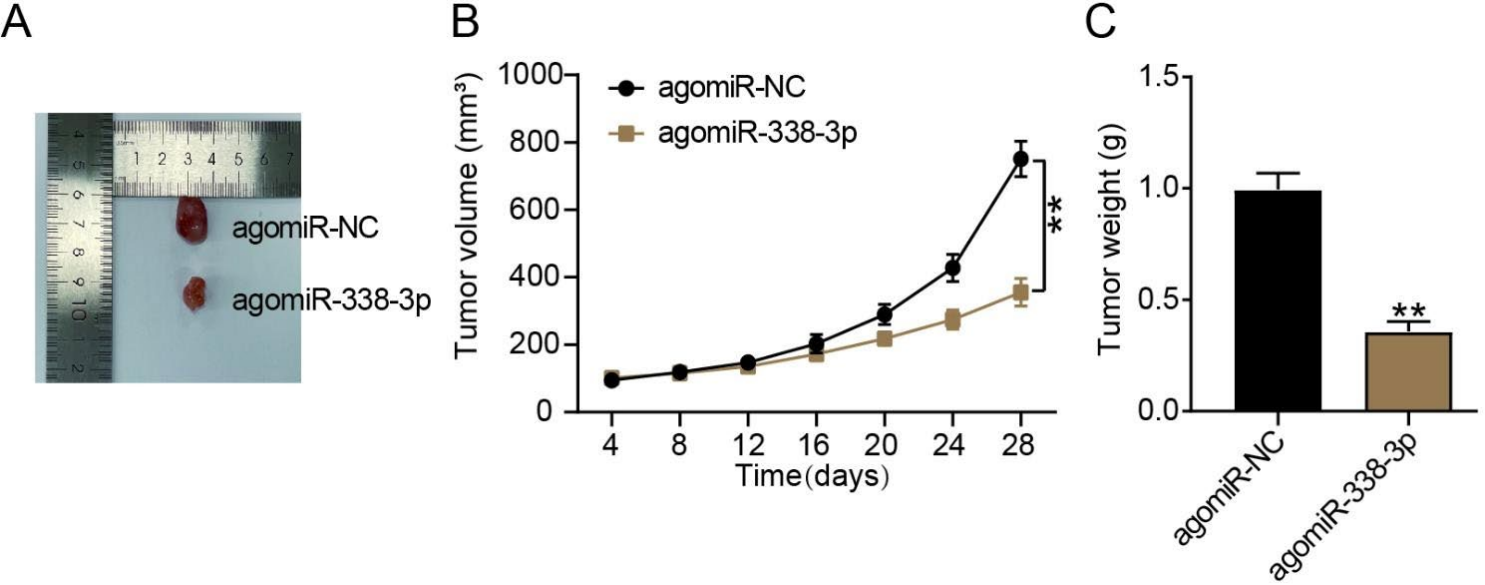
miR-338-3p overexpression inhibited glioma tumor growth in vivo. Mice were injected with 2 × 10^7^ agomiR-338-3p/NC-transfected U251 cells. The mice were observed every 4 days. On day 28, the mice were sacrificed, and their tumors were collected. (**A**) Representative pictures of tumors in each group. (**B, C**) The volume (**B**) and weight (**C**) of the tumors were measured in each group. ^**^P < 0.001 vs. agomiR-NC

To further confirm how miR-338-3p affects the development of glioma tumors, in vivo experiments were performed. Tumors in the agomiR-338-3p group were smaller than those in the agomiR-NC group (Fig. 2A). AgomiR-338-3p significantly inhibited tumor growth 28 days after injection; hence, the agomiR-338-3p group manifested a smaller tumor volume and weight than did the agomiR-NC group (*P* < 0.001; Fig. 2B and 2C). Therefore, we revealed that miR-338-3p overregulation impeded glioma tumor growth in vivo.

### Mir-338-3p targeted THBS1

**Fig. 3:**
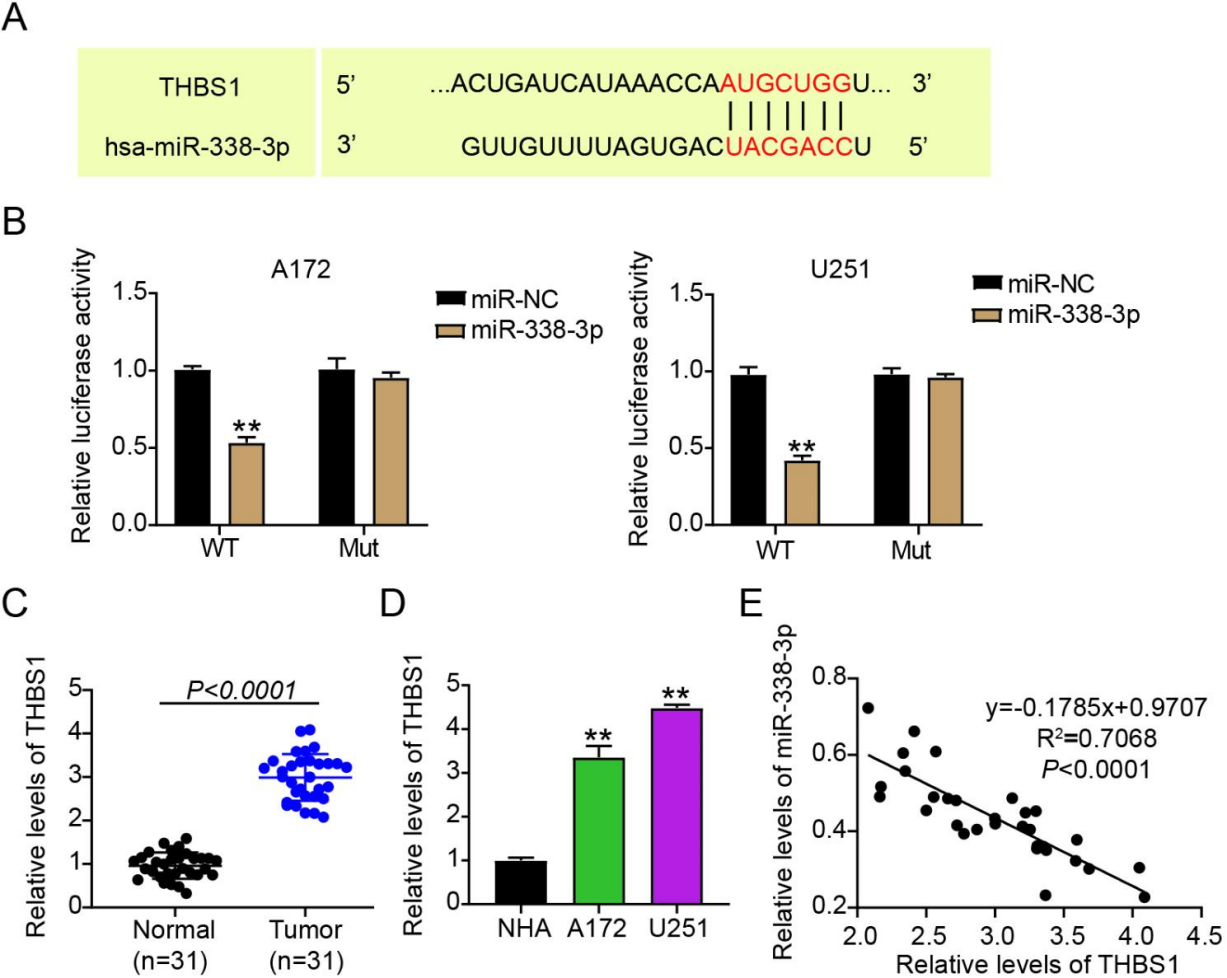
THBS1 was targeted by miR-338-3p.(**A**) TargetScan was used to predict THBS1 binding sites on miR-338-3p. (**B**) Dual-luciferase activity analysis was performed to confirm the relationship between miR-338-3p and THBS1. ^**^P < 0.001 vs. miR-NC. (**C**) THBS1 expression in normal and glioma samples was assessed. (**D**) THBS1 expression in primary normal human astrocytes (NHA) and glioma cells (U251 and A172) was assessed. ^**^P < 0.001 vs. NHA. (**E**) Spearman’s correlation analysis showed the correlation between THBS1 expression and miR-338-3p expression in glioma tissues

miRNA targets mRNA and regulates cancer progression. Herein, miR-338-3p target gene candidates were identified using TargetScan. THBS1 contained miR-338-3p binding sites (Fig. 3A). Hence, we verified the role of miR-338-3p in THBS1 targeting by performing dual-luciferase analysis. miR-338-3p overregulation considerably reduced the luciferase activity of WT-THBS1; however, it barely affected the activity of Mut-THBS1 (*P* < 0.001; Fig. 3B). We subsequently assessed THBS1 levels in the clinical samples and glioma cell lines. THBS1 was notably upregulated in tumor tissues and A172 and U251 cells compared with that in normal tissues and NHA cells, respectively (*P* < 0.001; Fig. 3Cs and 3D). In gliomas, miR-338-3p expression was negatively correlated with THBS1 expression, as determined by Spearman’s test (R^2^ = 0.7068; *P* < 0.0001; Fig. 3E). In summary, THBS1 was overexpressed in gliomas and was targeted by miR-338-3p.

### Overregulated mir-338-3p targeting THBS1 attenuated glioma progression by suppressing the PI3K/Akt pathway

**Fig. 4:**
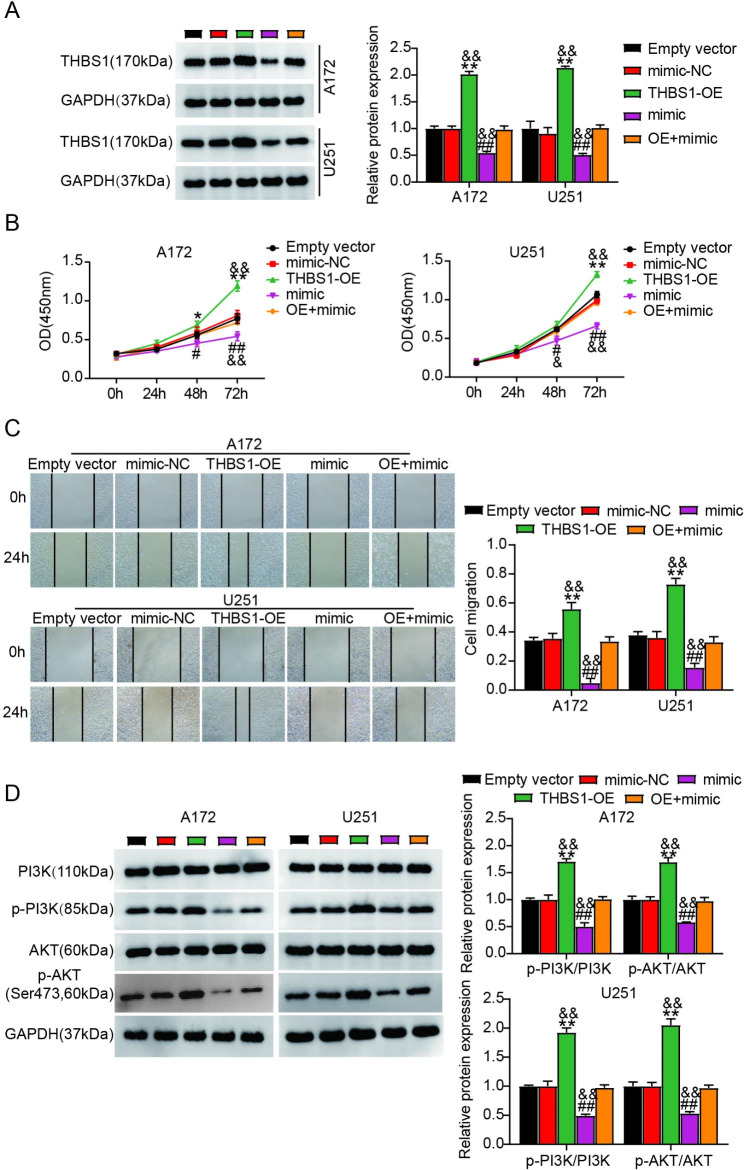
Overregulated miR-338-3p targeting THBS1 attenuated glioma cell growth by suppressing the PI3K/Akt pathway. (**A**) The western blot analysis detected THBS1 levels in U251 and A172 cells transfected with mimic-NC, empty vector, THBS1-OE (OE), miR-338-3p mimic (mimic), or OE + mimic. (**B, C**) Proliferation (**B**) and migration (**C**) of U251 and A172 cells transfected with mimic-NC, empty vector, OE, mimic, or OE + mimic were evaluated by performing CCK-8 and wound-healing analyses. (**D**) Levels of phosphorylated AKT and PI3K proteins in U251 and A172 cells harboring mimic-NC, empty vector, OE, mimic, or OE + mimic were assessed by performing western blot analysis. ^*^P < 0.05, ^**^P < 0.001 vs. Empty vector; ^#^*P* < 0.05, ^##^*P* < 0.001 vs. mimic-NC; ^&^*P* < 0.05, ^&&^*P* < 0.001 vs. OE + mimic.

We further investigated the function of THBS1 and its regulatory relationship with miR-338-3p in gliomas. The western blotting analysis revealed that THBS1 expression was upregulated in A172 and U251 cells transfected with THBS1-OE, and this change was reversed by transfecting the miR-338-3p mimic into U251 and A172 cells (Fig. 4A). We assessed how THBS1 affected the migration and proliferation of U251 and A172 cells in vitro. Transfection with THBS1-OE significantly promoted the proliferation and migration of glioma cells (*P* < 0.001; Fig. 4B and 4C). Moreover, THBS1 overexpression restored the inhibitory role of miR-338-3p in the migration and proliferation of glioma cells. As for the PI3K/Akt pathway, THBS1 overexpression reduced the effect of the miR-338-3p mimic on the expression of p-Akt (Ser473) and p-PI3K by significantly downregulating them (Fig. 4D). These results show that overregulated miR-338-3p targets THBS1, thereby attenuating glioma progression by inhibiting the PI3K/Akt pathway.

## Discussion

**Fig. 5:**
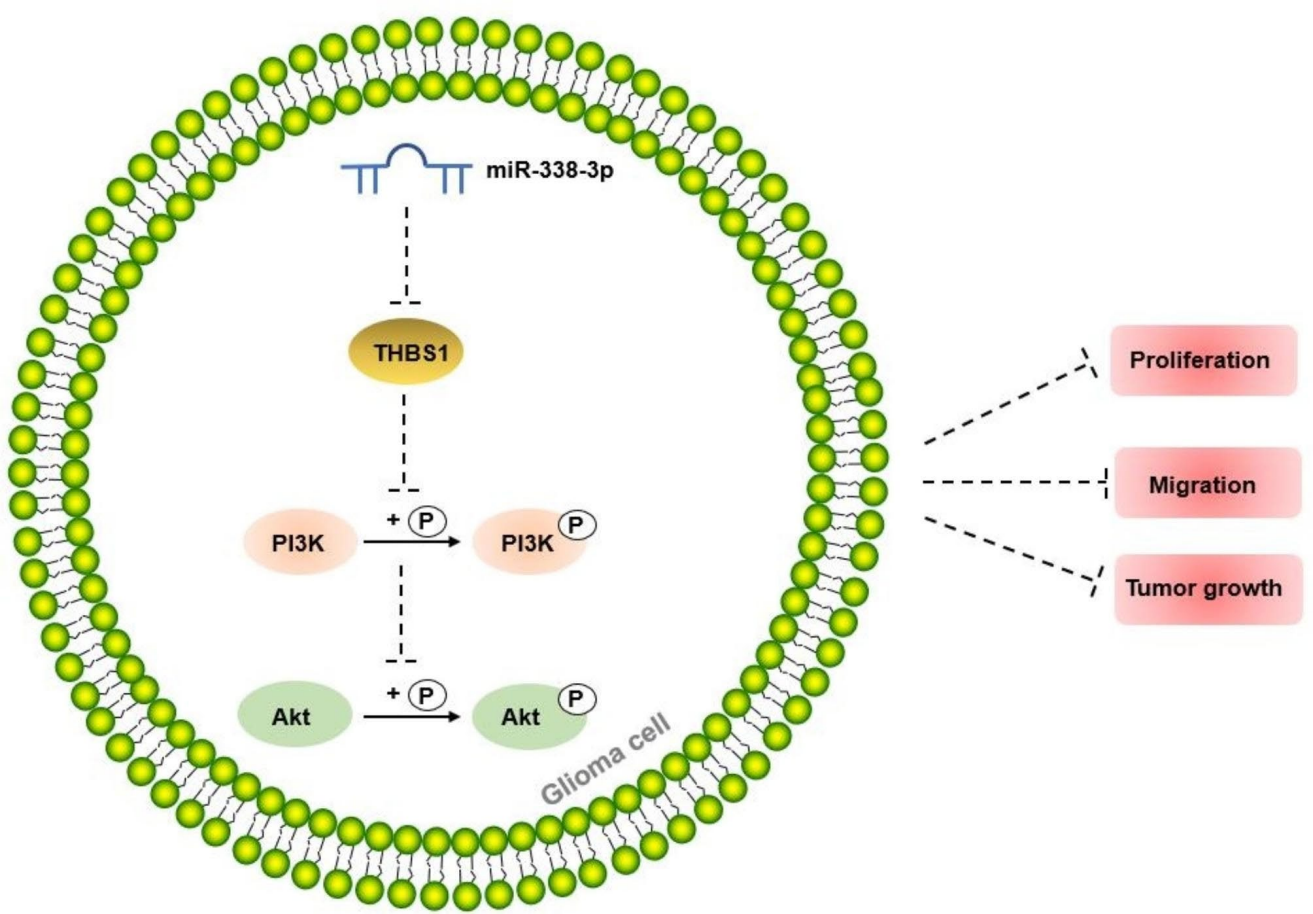
miR-338-3p attenuated glioma progression by inhibiting THBS1 to inactivate the PI3K/Akt pathway

Gliomas are brain tumors with high morbidity, mortality, and poor prognosis [19]. Effective prevention, diagnosis, and treatment using biomarkers play important roles in cancers, including gliomas. miRNAs have been reported to be involved in glioma regulation. miR-338-3p is a putative tumor suppressor in several cancers, including glioma. However, its role in glioma remains unclear; hence, we examined its molecular mechanism of action in glioma progression. We found that miR-338-3p was downregulated in gliomas, and its overexpression was associated with the inhibition of cell growth. Furthermore, targeting THBS1 by miR-338-3p overexpression attenuated glioma progression by blocking the PI3K/Akt pathway (Fig. 5).

miR-338-3p downregulation has been detected in several cancers [20–24] and has been documented to be an essential tumor suppressor. Overregulated miR-338-3p targets corresponding relevant genes to repress the migration, invasion, and proliferation of glioma cells. For example, miR-339-3p targets metastasis associated in colon cancer 1 to induce glioma cell apoptosis and enhance glioma cell proliferation [25]. miR-339-3p targets smoothened and suppresses the proliferation, migration, and invasion of glioma cells [26]. The present results are consistent with those of previous studies, showing that the low expression of miR-339-3p was observed in glioma and tumorigenesis was inhibited. Additionally, we identified a new target gene of miR-338-3p, THBS1, in gliomas, indicating that miR-338-3p plays a role in gliomas by targeting THBS1.

THBS1 promotes tumor progression and metastasis by regulating cell migration, proliferation, angiogenesis, and apoptosis [27–29]. Previous studies have shown that THBS1 inhibition effectively prevents glioma tumorigenesis [12, 30]. The present study revealed that THBS1 overexpression enhanced the proliferation and migration of glioma cells, suggesting an oncogenic role of THBS1 in glioma. Lin et al. [31] demonstrated that miR-338-3p reduced oxaliplatin resistance by targeting THBS1 in gastric cancer cells. Wei et al. [32] confirmed the targeting role of THBS1 on miR-338-3p in patients with hepatocellular carcinoma and showed a highly significant diagnostic value. However, the relationship between miR-338-3p and THBS1 in glioma remains unclear. Here, we demonstrated, for the first time, the targeting role of miR-338-3p on THBS1, showing that miR-338-3p exerted its antitumor effect on glioma by targeting THBS1. As for the downstream of THBS1, we also found that the PI3K/Akt pathway was activated by THBS1 overexpression.

The PI3K/Akt pathway is predominantly responsible for THBS1 upregulation [33]. PI3K/Akt plays a role in proliferation and migration by inducing endothelial growth factors [34, 35]. Recently, PI3K/Akt has been shown to play a role in the molecular mechanisms of cancer. The activated PI3K/Akt pathway promotes cell motility and migration by decreasing adhesion to uveal melanoma cells [36]. In other studies on glioma cells, the PI3K/Akt pathway has been shown to modulate the migration, invasion, and proliferation of tumor cells [37–39]. Liao et al. [37] demonstrated that fibronectin-1 activated the PI3K/Akt pathway and stimulated glioma growth and invasion. In a treatment analysis of gliomas, plumbagin was considered a potential anti-invasive agent that silenced the PI3K/Akt pathway, suggesting that the PI3K/Akt pathway could be used as a therapeutic biomarker [38]. Here, we confirmed for the first time that miR-338-3p targeting THBS1 reduced the levels of p-PI3K and p-Akt in glioma cells, indicating that miR-338-3p targeting THBS1 inhibited the activation of the PI3K/Akt pathway in glioma.

This study revealed that miR-338-3p prevented glioma tumorigenesis by targeting THBS1 to inhibit the activation of the PI3K/Akt pathway. However, this study has several limitations. First, the mechanism through which the PI3K/Akt pathway is involved in gliomas requires further elucidation. Second, the direct relationship between THBS1 and the PI3K/Akt pathway needs to be confirmed. Third, animal experiments verifying the regulatory mechanisms of the THBS1 and PI3K/Akt pathways are lacking. The regulatory axis is a complex process requiring further investigation. We will address these issues further in the follow-up period.

## Conclusion

In conclusion, we revealed the downregulation of miR-338-3p in glioma. This may abate the migratory and proliferative capacities of glioma cells by modulating the PI3K/Akt/THBS1 axis. The results of this study corroborate the involvement of miR-338-3p in glioma progression. Additionally, these results highlight miR-338-3p as a promising biomarker for glioma.

**Declarations**.

### Electronic supplementary material

Below is the link to the electronic supplementary material.

Supplementary Material 1

## Data Availability

All data generated/analyzed in this research have been appended in this article.
